# The Relationship between Self-Perceived Burden and Posttraumatic Growth among Colorectal Cancer Patients: The Mediating Effects of Resilience

**DOI:** 10.1155/2019/6840743

**Published:** 2019-09-12

**Authors:** Chengshuai Zhang, Ruitong Gao, Jiandong Tai, Yuewei Li, Si Chen, Lei Chen, Xiaobai Cao, Li Wang, Minghua Jia, Feng Li

**Affiliations:** ^1^School of Nursing, Jilin University, Changchun 130000, China; ^2^Shengli Oilfield Central Hospital, Dongying, China; ^3^The First Hospital of Jilin University, Changchun 130000, China

## Abstract

At present, the influence factors of posttraumatic growth (PTG) in colorectal cancer (CRC) patients and the relationship between PTG, self-perceived burden (SPB), and resilience are not completely clear. Thus, the present study examined whether resilience and SPB could predict PTG in CRC patients. The role of resilience as a potential mediator was also assessed. Using a cross-sectional design, a convenience sample of 157 CRC patients was selected as subjects, from July to December 2016 in a third-grade hospital. It was found that the main influencing factors for the total PTG score of CRC patients included work status, affordability for medical expenses, and duration of illness. Resilience was positively correlated with PTG, SPB was negatively correlated with PTG, and resilience played an intermediary role. Our findings remind clinicians to treat the psychosocial response of CRC patients from multiple perspectives, with a focus on their positive aspects. By increasing resilience and reducing the patient's SPB, clinicians might enhance the patient's PTG and quality of life.

## 1. Introduction

Colorectal cancer (CRC) is the third most common global cancer and a leading cause of cancer-related death [1]. Over 1.2 million new CRC cases are reported each year, and 600,000 patients die of this disease. Due to changes in living standards and the dietary patterns of Chinese residents, the incidence of CRC has increased year by year and in some developed regions the reported number of cases exceed those of high incidence countries [2, 3].

Cancer patients have concerns over their survival time, treatment side effects, and recurrence. These problems increase cancer-related mental problems including anxiety or depressive symptoms, interpersonal stress, and loneliness [4, 5], but some studies suggest that most CRC patients experience positive changes [6–8]. Posttraumatic growth (PTG) describes the positive changes that individuals experience after stressful events [9, 10]. Positive PTG can help cancer patients cope with cancer-related psychological stress, and so research on PTG would promote the adaptation, growth, and recovery of patients after the traumatic event of cancer.

SPB, as a negative psychological experience, refers to the psychological feelings of guilt, frustration, and anxiety when patients believe they are becoming a burden to their family and others [11]. Studies have shown that cancer patients have high levels of SPB and PTG [12, 13].

Resilience, also known as resistance or psychological resilience, refers to the ability of individuals to successfully cope and adapt to difficulties and is one of the most commonly used indicators for evaluating positive psychological conditions [14, 15]. As resilience represents a dynamic, changeable path that can raise the level of hope and positive attitude, so active interventions based on resilience might represent a favorable choice for cancer patients [16].

This study therefore proposes a correlation between SPB, resilience, and PTG. Resilience may represent a mediator of SPB that affects PTG. Hence, we aimed to provide a basis for clinical caregivers to intervene in CRC patients through understanding the relationship between the three variables and select better and more appropriate intervention opportunities and variables.

## 2. Methods

### 2.1. Subjects

A cross-sectional, observational study was conducted in a third-grade hospital in a northeast city in China from July to December 2016. Inclusion criteria were as follows: (1) age ≥18 years old; (2) primary colorectal cancer diagnosed by pathology; (3) voluntary participation with informed consent. Exclusion criteria were as follows: (1) cognitive difficulties, difficulty in reading and completing questionnaires; (2) being in a critical condition without the ability to complete the study.

This study was approved by the ethics committee of the author's academy. All questionnaires were collected by trained research assistants. Prior to the investigation, patients were informed the purpose of the study, their voluntary participation, and the right to withdraw from the study at any stage. Participants signed informed consent forms prior to study initiation and filled out the questionnaire during their hospitalization.

### 2.2. Research Tools

#### 2.2.1. General Information Questionnaire

The general information questionnaire consisted of 13 items, including age, gender, marital status, and education level.

#### 2.2.2. Posttraumatic Growth Inventory-Chinese Version

The Posttraumatic Growth Inventory (PTGI) [14] was used to assess the degree of positive psychological changes after the individual has experienced a traumatic event, including five dimensions: (1) relating to others, (2) new possibilities, (3) personal strength, (4) spiritual change, and (5) appreciation of life, totaling 21 items. We applied the Chinese revised version of PTGI (C-PTGI) [17]. The Likert 6 used by the C-PTGI, ranged from “no posttraumatic changes” (0 points) to “very large posttraumatic changes” (5 points), with a total score of 0 to 100 points. A higher score indicated a more positive psychological experience. In this study, Cronbach's coefficient of the C-PTGI was 0.918, indicating a good reliability and validity.

#### 2.2.3. Self-Perceived Burden Scale

The self-perceived burden scale (SPBS) included three dimensions of body burden, emotional burden, and economic burden, consisting of 10 items [18]. Cronbach's *α* score was 0.85 [19]. The SPBS score adopted a Likert 5 rating, from “never” (1 point) to “always” (5 points), with a total score that was either positive or negative (only the eighth item was scored in reverse, the others were positive scores). A higher total score indicates a higher level of individual SPB.

#### 2.2.4. Connor-Davidson Resilience Scale

The Connor-Davidson Resilience Scale (CD-RISC) consisted of three dimensions: tenacity, strength, and optimism, totaling 25 entries. Cronbach's *α* coefficient was 0.93 [20].

### 2.3. Statistical Analysis

We used the Epidata 3.1 to establish a database and SPSS 21.0 for statistical analysis. The general information of CRC patients was analyzed by descriptive statistical analysis of frequency and percentage. The status of PTG was statistically described by the mean and standard deviation. Univariate analysis and multiple stepwise regression were used to analyze the influencing factors of PTG. A Pearson or Spearman correlation analysis was performed to assess the relationship between PTG and SPB. The structural equation model was constructed using AMOS.

## 3. Results

### 3.1. Participant General Information

Considering the number of entries in C-PTGI used in this survey was 21 and the sample loss of 10%~20%, in order to ensure sufficient sample size, a total of 200 questionnaires were distributed [21]. Finally, 190 questionnaires were collected, and incomplete questionnaires or all entries with the same score were considered invalid and excluded.

Overall, 157 questionnaires were accepted and the questionnaire response efficiency was 78.5%. Among these, 49% of participants were over 60 years old, more males (58.6%). The vast majority of participants were married (95.5%) and currently lived with their spouses (75.2%). CRC treatments were mainly surgery with radiotherapy or chemotherapy (70.7%), and the duration of illness lasted from one month to one year (70.1%). Specific data is shown in Table 1.

### 3.2. Scores of PTG, SPB, and Resilience

The mean score of PTG was 76.78 (SD = 14.98; range 22-100), with a mean score per item of 3.84 (on a 0-5 scale), which reflected an upper-moderate level of PTG among the CRC patients. The mean score of SPB was 34.81(SD = 6.82; range 19-46), with a mean score per item of 3.48 (on a 1-5 scale), which reflected a moderate level of SPB among the CRC patients. The mean score of resilience was 69.03 (SD = 19.06; range 18-100), with a mean score per item of 2.761 (on a 0-4 scale), which reflected a relatively low level of resilience among the CRC patients. Means and standard deviation of all variables regarding PTG, SPB, and resilience are shown in Table 2.

### 3.3. Analysis of the Factors Influencing PTG in CRC Patients

The results revealed significant differences (*P*<0.05) in age, work status, economic affordability, therapy method, illness time, and living arrangements. Table 1 shows specific data. The results of multivariate analysis showed that work status, economic affordability, and duration of illness were the main factors affecting PTG in CRC patients (Table 3).

### 3.4. Correlations among PTG, SPB, and Resilience in CRC Patients

The results showed that the total PTG scores were negatively correlated with the total SPB scores (*r*=-0.21,* P*<0.01) and other dimensions were negatively correlated excluding body burden and the appreciation of life. The total and each dimension scores of SPB were negatively correlated with resilience. There is a positive correlation between PTG and resilience (*r*=0.73,* P*<0.01). Specific results are shown in Table 2.

### 3.5. Path Analysis of SPB in the Process of PTG Affected by Resilience

#### 3.5.1. Regression Analysis of SPB on PTG

The results of regression analysis showed* R*^*2*^ of 4%, indicating that SPB could explain the variance of 4% in the PTG. The specific results are shown in Table 4.

#### 3.5.2. Regression Analysis of SPB on Resilience

The results of regression analysis showed* R*^*2*^ of 11.3%, indicating that SPB could explain the variance of 11.3% in the resilience. The specific results are shown in Table 5.

#### 3.5.3. Regression Analysis of Resilience on PTG

The results of regression analysis showed* R*^*2*^ of 53.2%, indicating that resilience could explain the variance of 53.2% in the PTG. The specific results are shown in Table 6.

#### 3.5.4. Regression Analysis of SPB and Resilience on PTG

The results of regression analysis showed* R*^*2*^ of 53.4%, indicating that SPB and resilience could explain the variance of 53.4% in the PTG. At the same time, the SPB showed no effect on PTG. The specific results are shown in Table 7.

The SPB was found to influence the PTG level of CRC patients by affecting the level of resilience. The standardized path coefficient was c=-0.210 (*P*=0.008). From regression analysis, the impact of SPB on resilience using the standardized regression of coefficient a=-0.337 (*P*≤0.001) showed a significant difference. From the regression analysis of the impact of resilience on PTG, the path coefficient of b=0.730 (*P*≤0.001) reached a significant level, and thus resilience affected PTG. The SPB and resilience were simultaneously included when calculating the regression of PTG. The path coefficient of c′=0.040 did not show a significance influence (*P*=0.492), indicating that the effects of SPB on PTG were through resilience. Resilience therefore played an intermediary role in the process of SPB affecting PTG (Table 8).

### 3.6. Path Effect Decomposition

**Figure 1:**
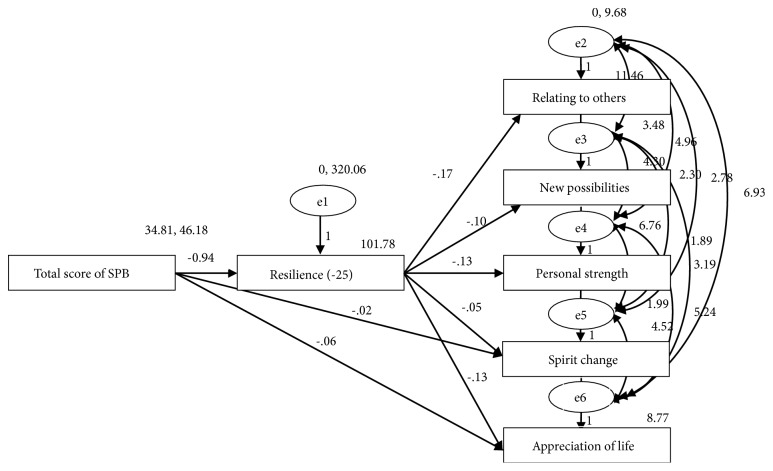
Initial model fitting and model modification.

We constructed and fitted the initial theoretical model which was amended using the model correction index (MI). The revised model is shown in Figure 1. The fit results showed that* x*^*2*^*/df*=0.48<2(*P*=0.315), other indicators such as GFI, AGFI, NFI, TLI, CFI, and IFI were greater than 0.95, and RMSEA <0.05, indicating that the model had a good fit and was more reasonable (Table 9).

By decomposing the path coefficients, we found that the SPB had no direct effect on the other dimensions of PTG, except for the spiritual change. The results showed that the effects of the SPB to PTG were through resilience. We observed a direct influencing spiritual change and the appreciation of life, but the direct action path coefficient of the appreciation of life dimension was not significant (*P*=0.10), indicating that the influence was through the indirect effects of resilience. The effects of SPB on each dimension of PTG were spiritual change -0.260, new possibilities -0.231, appreciation of life -0.112, relating to others -0.244, and personal power -0.240 (Table 10).

## 4. Discussion

This study aims to explore the relationship between PTG, SPB and resilience in colorectal cancer patients. The results showed that PTG was negatively correlated with SPB and positively correlated with resilience. In addition, resilience played a full mediating role in PTG and SPB in colorectal cancer patients.

The PTG scores in this study were higher than scores in lung cancer, breast cancer, and colorectal cancer survivors with permanent intestinal ostomies [22–24]. This may be due to the continuous improvement of the cure rate and survival rate of colorectal cancer, which might influence their perception of positive findings. Moreover, none of the colorectal cancer survivors in our study underwent colostomy; after all, ostomy is a permanent injury for patients, which inevitably affects body function and changes body image, leading to more negative emotions.

Multivariate analysis showed that PTG was associated with work status, affordability for medical expenses, and duration of illness. Regard work status, in current study we found the retirees have the highest PTG, in line with the research of Wang et al. [25]. Firstly, retirees are older; their understanding of life and themselves may be more comprehensive and profound, the more mature and effective coping styles which may lead to more positive psychological changes [26]. Secondly, the retirees have relatively free time, so they would spend more time in receiving treatment [27]. Thirdly, the retired people do not have to deal with the pressure and burden from work and have less concern about the impact of the disease on future work. Consistent with previous studies, this study also found that individuals who can afford medical expenses have higher posttraumatic growth levels [25, 28, 29]. This may be because they have less concern about economic problems and more resources [30]; they can afford more expensive drugs or treatment. Patients feel less burden, negative emotions, and disease uncertainty, thus promoting their PTG. Patients with shorter duration of illness in this study have higher PTG. It may be because, with the prolonged illness, the patient suffers from the pain, gradually loses confidence, and becomes more worried about the treatment or prognosis. At the same time, high medical expenses and the use of social resources also lead to the increase of patients' SPB to some extent, resulting in sense of guilt and negative emotions, which further leads to the decline of PTG. However, in some studies of breast cancer patients, higher PTG was found in patients with longer disease duration [31, 32]. This may be caused by disease specificity.

Previous studies have shown a correlation between PTG and negative psychological status. The level of PTG was significantly correlated with the painful condition of head and neck cancer and breast cancer patients and was significantly negatively correlated with depression and anxiety [33, 34]. Perceived stress was also negatively associated with PTG (*r *= 0.36,* P *<0.001) [35]. Consistent with previous studies, SPB, as a negative psychological experience, was negatively correlated with PTG in colorectal cancer patients.

In keeping with previous studies, this study also found individuals with better resilience experience would have a higher degree of growth after traumatic events [24, 36, 37]. Resilient individuals are more likely to appraise adverse situations as challenges and exhibit cognitive flexibility, which contribute to the positive changes.

This study demonstrates that the effect of SPB on PTG is entirely through the resilience, which is consistent with the findings of the role of resilience in PTG and ruminating [36]. The mediating effect of resilience provides a new perspective for improving PTG in colorectal cancer patients. Resilience as a factor that can be adjusted in multiple ways, for example, from social-emotional training and training in cognitive reappraisal, clinicians should use it appropriately to improve health outcomes [38–40].

This study has several limitations. Firstly, it was a cross-sectional study, a causal relationship cannot be inferred, and it cannot provide information on dynamic changes of PTG after colorectal cancer. Secondly, the sample size in this study was relatively small, only limited to the third-grade hospitals in a city in China. Thirdly, the effect of the treatment method on patients' psychology was not considered, such as the side effects of radiotherapy and chemotherapy. Therefore, the results had specific limitations. In future work, it is necessary to expand the sample size and scope, perform follow-up reports from the initial diagnosis of cancer patients, analyze dynamic changes of PTG levels in various periods, and comprehensively integrate the relevant factors of PTG to form a high level theoretical framework.

## 5. Conclusion

On the above, there is a correlation between SPB, resilience, and PTG in colorectal cancer patients; the level of SPB and resilience has a certain predictive effect on PTG. Resilience plays a fully intermediary role in the impact of SPB on PTG. In the future, it is necessary to study the methods to improve PTG and resilience in colorectal cancer patients.

## Figures and Tables

**Table 1:** Participants' characteristics and differences of the PTG in different groups (N = 196).

Characteristic	Category	N(%)	Mean ± SD	*F/t/z/χ* ^2^	*P*
Age	18~40	17(10.8)	78.12±16.85	8.753^a^	0.033*∗*
	41~50	30(19.1)	71.77±13.30		
	51~60	33(21.0)	79.67±19.58		
	>60	77(49.0)	77.21±12.56		
Gender	Male	92(58.6)	76.57±15.07	-0.217	0.829
	Female	65(41.4)	77.09±14.96		
Marital status	Married	128	77.26±15.17	4.333^a^	0.115
	Single	7	66.80±3.35		
	Divorced/widowed	22	74.14±14.28		
Work status	Retirement	66(42.0)	82.56±11.17	18.902^a^	≤0.001*∗∗*
	On job	43(27.4)	73.65±19.71		
	Others	48(30.6)	71.65±11.92		
	(Dimission/suspension)				
Education	Less than high school	80 (51.0)	78.86±12.52	1.775	0.078
	High school and above	77(49.0)	74.62±16.98		
Medical expenses payment method	Medical insurance	143(91.1)	76.14±11.90	-0.205	0.840
	Self-paying	14(8.9)	76.85±15.28		
Residence	Urban	98(62.4)	77.14±15.82	1.037	0.301
	Rural	59(37.8)	75.19±13.45		
Affordability for	Yes	119(75.8)	79.32±15.55	5.495^a^	≤0.001*∗∗*
medical expenses	No	38(24.2)	68.25±8.62		
Religion	Yes	8(5.1)	77.25±15.88	0.090	0.928
	No	149(94.9)	76.76±14.99		
Income (RMB)/ month	<¥1000	27(17.2)	75.22±13.19	0.215	0.807
	¥1000~5000	101(64.3)	77.31±14.27		
	>¥5000	29(18.5)	76.41±18.91		
Therapy method	Radiation+ Chemotherapy	32(20.4)	75.06±14.63	7.290	0.001*∗∗*
	Surgery+ Radiation/Chemotherapy	111(70.7)	78.94±13.98		
	Surgery + Radiation + Chemotherapy	14(8.9)	63.64±17.18		
Duration of illness	≤1 month	30(19.1)	80.40±13.72	4.442	0.013*∗∗*
	1 month ~1 year	110(70.1)	77.25±14.63		
	>1 year	17(10.8)	67.41±16.37		
Living arrangement	Live alone	10(6.4)	66.50±14.63	3.321	0.039*∗∗*
	Live with spouse	118(75.2)	78.21±15.38		
	Live with children	29(18.5)	74.52±11.90		

*∗P*<0.05; *∗∗P*<0.01, statistically significant.

**Table 2:** Means, standard deviations, and correlations among PTG, SPB, and resilience (N = 196).

Variable	Mean ± SD	Range	1	2	3
1. PTG	76.78±14.98	22~100	1	-0.21*∗∗*	0.730*∗∗*
2. SPB	34.81±6.82	19~46		1	-0.337*∗∗*
3. Resilience	69.03±19.06	18~100			1

*∗∗P*<0.01, statistically significant.

**Table 3:** Multivariate analysis of PTG in colorectal cancer patients.

Variable	*B*	*Beta*	*t*	*P*
Constant	113.611		12.675	≤0.001
Work status	-4.350	-0.246	-3.130	0.002
Duration of illness	-6.257	-0.227	-3.081	0.002
Affordability for medical expenses	-8.953	-0.252	-3.187	0.002

*R* ^2^=0.216, adjusted *R*^2^=0.185, and *F*=6.901.

**Table 4:** Regression analysis of SPB on PTG.

Variable	*B*	*Beta*	*t*	*P*
Constant	92.836		15.171	≤0.001
SPB	-0.461	-0.210	-2.673	0.008

*R* ^2^=0.044, adjusted *R*^2^=0.038, and *F*=7.143.

**Table 5:** Regression analysis of SPB on resilience.

Variable	*B*	*Beta*	*t*	*P*
Constant	101.780		13.571	≤0.001
SPB	-0.941	-0.337	-4.449	≤0.001

*R* ^2^=0.113, adjusted *R*^2^=0.108, and *F*=19.795.

**Table 6:** Regression analysis of resilience on PTG.

Variable	*B*	*Beta*	*t*	*P*
Constant	37.198		12.036	≤0.001
Resilience	0.573	0.730	13.285	≤0.001

*R* ^2^=0.532, adjusted *R*^2^=0.529, and *F*=176.495.

**Table 7:** Regression analysis of SPB and resilience on PTG.

Variable	*B*	*Beta*	*t*	*P*
Constant	33.389		5.265	≤0.001
Resilience	0.584	0.743	12.721	≤0.001
SPB	0.088	0.040	0.688	0.492

*R* ^2^=0.534, adjusted *R*^2^=0.528, and *F*=88.185.

**Table 8:** Path analysis of SPB in the process of PTG affected by resilience.

	Standardized regression equation	Regression coefficient test
Step 1	Y=-0.210	SE=0.173, t=-2.673*∗∗*
Step 2	M=-0.337	SE=0.211, t=-4.449*∗∗*
Step 3	Y=0.040+0.743 M	SE=0.128, t=0.688
		SE=0.046, t=12.721*∗∗*

*∗∗*P<0.01.

**Table 9:** Path model fitting index.

	CMIN/DF	GFI	AGFI	NFI	TLI	CFI	IFI	RMSEA
This model	1.183	0.994	0.988	0.996	0.996	0.999	0.999	0.034
Standard	<2	>0.95	>0.95	>0.95	>0.95	>0.95	>0.95	<0.05

**Table 10:** Model path effect decomposition.

Effect	Independent variable	Standardized direct effect	Standardized indirect effect	Total standardized effect
Resilience	Total SPB scores	-0.337	0.000	-0.337
Spiritual change	Resilience	0.519	0.000	0.519
New possibilities	Resilience	0.686	0.000	0.686
Appreciation of life	Resilience	0.615	0.000	0.615
Relating to others	Resilience	0.726	0.000	0.726
Personal strength	Resilience	0.713	0.000	0.713
Spiritual change	Total SPB scores	-0.86	-0.175	-0.260
New possibilities	Total SPB scores	0.000	-0.231	-0.231
Appreciation of life	Total SPB scores	0.095	-0.207	-0.112
Relating to others	Total SPB scores	0.000	-0.244	-0.244
Personal strength	Total SPB scores	0.000	-0.240	-0.240

## Data Availability

The data used to support the findings of this study are available from the corresponding author upon request.
